# Do outcome measures used in neurological clinical research realistically represent the needs and the expectations of patients and their care givers?

**DOI:** 10.1186/1745-6215-16-S1-P8

**Published:** 2015-05-29

**Authors:** MG Celani, MC Bassi, A Bignamini, P Candelaresi, M Carlini, M Cecconi, M Congedo, C Cusi, S Cuzzubbo, D Guerra, S Macone, M Melis, C Motto, K Nardi, V Oppo, R Papetti, C Piersanti, V Piras, A Serafini, AL Sgoifo, E Susani, L Tremolizzo, TA Cantisani

**Affiliations:** 1 Azienda Universitaria Ospedaliera di Perugia, Perugia, Italy; 2 Cochrane Neurological Field - Health Authority of Umbria – Perugia, Italy; 3 Agenzia sanitaria e sociale regionale - Regione Emilia-Romagna, Bologna, Italy; 4 Dpt of Pharmaceutical Sciences (DISFARM) - University of Milan – Milan, Italy

## Background

There is an increasing recognition that Neurological diseases, both chronic and disabling, are effected by multiple domains. It is crucial to select outcome measures and their rating scales to assess the meaningful results of a clinical study. The aim of the study is to address the mismatch between what clinical researchers do and what patients need.

## Methods

Stroke, dementia and epilepsy are the greater and most common disabling neurological diseases and were considered in this study. For each mentioned disease we will perform a Systematic reviews of all randomized clinical trials published in any language over the last 5 years with the aim of identifying and analyzing the outcome measures used in the evaluation of any kind of intervention. Fifteen Neurologists are filling out a single computerized form for each single trial assigned.

The form has the following information: *Characteristics* of the trial with relevance on quality, *Extent of variability in end points* selected and their domains, *Scales or techniques* used to make the measurement, type of analysis applied and time of measurement, *Presence of attrition bias* in terms of fraction of patients reported by end points on the number enrolled in the study, *Presence of outcome reporting bias* in terms of end points declared and non-reported, end points not declared but reported, *Source of funding.*

The valuation of patient and career needs and emotions will be performed with focus group discussions in a parallel section of the study.

## Results

**Figure 1:**
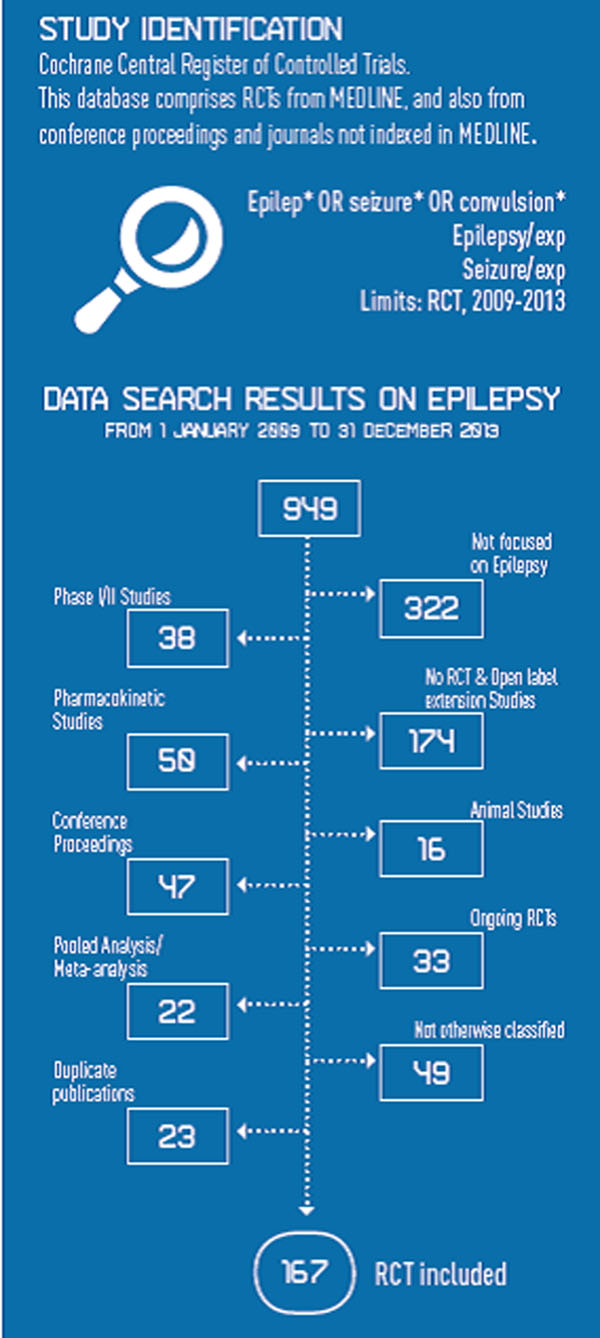
Trial flow diagram

We start to offer a photograph of recent research on Epilepsy. We searched electronic databases, the Cochrane Central Register of Controlled Trials (CENTRAL) and MEDLINE, as well as ongoing trial registries, from 1.01.2009 to 31.12.2013. We have examined 949 papers published (see trials flow diagram in Figure 1). Only 17.6% are randomized clinical trials while another 18% of these fail to reach the target on proven efficacy of evaluated treatment.

## Conclusions

This result gives a first indication of how valuable resources are wasted. More importantly we are considering the weaknesses in the design, analysis and conflicts of interest in the included 17.6% of RCTs, highlighting research that should be better directed.

This approach could provide useful information to clinicians, who will have an opportunity to learn how to use a new framework in the production, reporting and critical appraisal of literature and it could also help to bridge the gap between end-users and investigators and promote valuable clinical research.

